# Adult-Onset Still's Disease Masquerading as Sepsis in an Asplenic Active Duty Soldier

**DOI:** 10.1155/2012/349521

**Published:** 2012-11-25

**Authors:** Nathan T. Jaqua, David Finger, Joshua S. Hawley

**Affiliations:** ^1^Department of Internal Medicine, Tripler Army Medical Center, 1 Jarrett White Road Honolulu, HI 96859, USA; ^2^Department of Rheumatology, Tripler Army Medical Center, Honolulu, HI 96859, USA; ^3^Department of Infectious Disease, Tripler Army Medical Center, Honolulu, HI 96859, USA

## Abstract

This is a case of a 26-year-old active duty male with a history of idiopathic thrombocytopenic purpura (ITP) and surgical asplenia who presented with a one-week history of fevers, myalgias, arthralgias, and rigors. His evaluation upon presentation was significant for a temperature of 103 degrees F, white blood cell count of 36 K with a granulocytic predominance, and elevated transaminases. He was treated empirically with broad-spectrum antibiotics with concern for a systemic infection with an encapsulated organism. During his stay, he developed four SIRS criteria and was transferred to the progressive care unit for suspected sepsis. He continued to have twice-daily fevers and a faint, salmon-colored centripetal rash was eventually observed during his febrile episodes. After a nondiagnostic microbiologic and serologic workup, he was diagnosed with adult-onset Still's Disease and started on intravenous methylprednisolone with brisk response. He was discharged on oral prednisone and was started on anakinra. Adult-onset Still's disease is a rare condition that presents with varying severity, and this is the first reported case, to our knowledge, of its diagnosis in an asplenic patient. Its management in the setting of asplenia is complicated by the need for antibiotic therapy with each episode of fever.

## 1. Introduction

Adult-onset Still's disease (AOSD) is a rare systemic inflammatory illness that presents with nonspecific symptoms and may range in severity from mild to severe. It is a diagnosis of exclusion, often considered only after infectious etiologies have been excluded, and differentiating between sepsis and Still's may prove to be quite difficult in some cases. AOSD is of unknown etiology and is associated with spiking fevers, arthralgias or arthritis, evanescent rash, lymphadenopathy, hepatosplenomegaly, and myalgias. Accompanying symptoms of serositis (pleuritic or pericarditis) may also be present. There are reported cases of AOSD presenting as acute respiratory distress syndrome, liver or renal failure, thrombotic thrombocytopenic purpura, or aseptic meningitis [1, 2]. This case involves a 26-year-old very ill-appearing active duty male with a history of splenectomy secondary to ITP with a presentation concerning for severe sepsis. 

## 2. Case

A 26-year-old male with a history of splenectomy secondary to ITP refractory to steroids presented to the emergency department after a significant leukocytosis was found on laboratory analysis in the clinic. He had presented to the outpatient clinic complaining of 5 days of diffuse, severe myalgias and arthralgias. He initially attributed the symptoms to body-boarding which he participated in the day before symptom onset. In the outpatient clinic, he had a temperature of 101.5 degrees F and direct fluorescent antibody and a rapid group A streptococcus tests were negative; he was sent home with empiric oseltamivir and a presumptive diagnosis of influenza. The following day he again presented to the clinic, now with a temperature of 103.6 degrees F, and after laboratory evaluation revealed a white blood cell count of 32,600 with 87.8% granulocytes, he was instructed to proceed to the emergency department (ED). On review of systems, he reported nausea and vomiting and a mild sore throat at the start of the symptoms. From the ED he was admitted and started on vancomycin, ceftriaxone, and trimethoprim/sulfamethoxazole after initial blood cultures were obtained. Upon admission other laboratory abnormalities noted were elevated alanine aminotransferase (ALT) at 103 units/L, aspartate aminotransferase (AST) at 98 units/L, and alkaline phosphatase (ALP) at 171 units/L. Erythrocyte sedimentation rate was 46 mm/hr and C-reactive protein was 23.4 mg/dL. Platelets (PLT) were 374 K/L. Notably, fibrinogen was elevated, PLT were normal, and triglycerides were normal suggesting against macrophage activation syndrome. 

During the first two days of admission, the patient underwent a right upper quadrant ultrasound that was negative for cholelithiasis, cholecystitis, or choledocholithiasis, and he continued to endorse sore throat and diffuse myalgias and arthralgias. On day two of admission, he began complaining of both dyspnea and coughing with deep inspiration but denied pleuritic chest pain or hemoptysis. His SpO_2_ was 88–93% and attributed to hypoventilation from shallow breathing. Chest radiograph was unremarkable and there was no response to nebulizer treatments. On day five of his admission, he complained of sudden-onset dyspnea and pleuritic chest pain and was tachypneic and tachycardiac on exam. Vitals revealed a BP of 122/68, heart rate of 115, respiratory rate of 31, and temperature of 102.4 F. A chest radiograph demonstrated new atelectasis, and a subsequent CT pulmonary angiogram was negative for pulmonary embolism but did demonstrate bilateral pleural effusions. On day six of admission, the patient was transferred to a higher level of care to the progressive care unit (PCU) for continued dyspnea, pleuritic chest pain, oxygen requirement of 3 L NC, and 4 out of 4 SIRS criteria concerning for severe sepsis. 

Upon transfer and review of his records, it was noted by the medical team that thus far, negative workup included blood cultures, creatine kinase, ASO, and Monospot, as well as influenza A and B, adenovirus, RSV, parainfluenza, and metapneumovirus by direct fluorescent antibody. However, WBC, AST, ALT, and ALP all continued to be elevated as was CRP and ESR. Given the presentation doxycycline was started for atypical organisms and rickettsial coverage. It was also noted that during his hospitalization, the patient was having twice-daily fevers. Blood parasites were repeatedly checked and were negative for *Babesia* and malaria. A CT of the patient's abdomen and pelvis in search of an abscess, mass, or lymphadenopathy was nondiagnostic. 

Shortly after admission to the PCU during a febrile episode, it was observed that the patient developed an evanescent salmon-colored macular rash over his upper extremities. Further laboratory evaluation revealed a ferritin >10,000 ng/mL and elevated lactate dehydrogenase, consistent with Still's disease. Rheumatology consultation was obtained and therapy with corticosteroids was recommended. The patient had a marked response to systemic corticosteroids within the first 12 hours. He was eventually discharged home with anakinra, an IL-1 antagonist, with good response confirmed on outpatient rheumatology followup after tapering of steroids. 

## 3. Discussion

To establish the diagnosis of AOSD using the Yamaguchi criteria, five of the criteria should be fulfilled including two major criteria. Major criteria in our patient include a fever greater than 39 C (102.2 F) for at least one week, arthralgias (2 weeks or longer), salmon-colored rash over extremities noted during a febrile episode, and leukocytosis [3, 4]. He also demonstrated several minor criteria including sore throat, elevated liver enzymes and LDH, and negative rheumatoid factor. 

The nonspecific presentation of AOSD lends itself to a broad differential of infectious, inflammatory, and malignant etiologies. In rare cases it may even present masquerading as sepsis and cause abnormalities in multiple organ systems [2, 3]. It has been proposed that the use of biomarkers such as procalcitonin, C-reactive protein, and interleukin-8 may improve the diagnostic accuracy of infections and help differentiate from inflammatory conditions [5]. Many markers overlap between infectious and inflammatory conditions, but procalcitonin likely is a more sensitive and specific biomarker for bacterial infection [6]; however, there are limitations as fever associated with AOSD may mildly elevate PCT. Many cytokines have been measured and found to be elevated in AOSD including IL-1, TNF*α*, IL-6, and IL-18 [7]. However, none are specific enough to reliably differentiate sepsis from AOSD. 

In reviewing the literature, the theme for the diagnosis of AOSD and differentiating it from bacterial infection is reliance upon clinical manifestations, which is necessary since cytokine profiles alone are nonspecific and unreliable. One study in particular concluded that a modified Pouchot AOSD activity score was more useful in diagnosing AOSD than relying on biomarkers [5]. Regardless of the scoring system used, a high degree of suspicion must be maintained when patients present with systemic manifestations of severe illness, especially in light of continuing negative infectious evaluation, to reduce the delay of diagnosis which leads to appropriate treatment and prevents overuse of antibiotics and hospital resources. 

AOSD is usually treated with a combination of nonsteroidal anti-inflammatory drugs (NSAIDs), glucocorticoids, and methotrexate (MTX). In an asplenic patient, long-term corticosteroids are relatively contraindicated given the potential predisposition to infection, along with the other well-known undesirable side effects of long-term steroid treatment. MTX has been used successfully as a steroid-sparing agent. Alternatively, tumor necrosis factor inhibitors (TNFi) have also shown modest success in treating AOSD [8]. In our patient MTX was not ideal secondary to his elevated liver enzymes. Anakinra, an interleukin-1 (IL-1) inhibitor, was chosen for maintenance medication because of the shorter half-life and greater efficacy than TNFi. Given his predisposition to severe infection due to his asplenia, faster elimination once discontinued, if necessary, would be desirable. Anakinra has been shown to successfully treat AOSD [7, 9–14]. 

The first report of using anakinra was in patients with AOSD refractory to MTX, corticosteroids, and etanercept (TNFi) [8]. Prior to initiation of anakinra, the patients continued to have spiking fevers, synovitis, rash, elevated ferritin, WBC, and CRP on the standard therapy. Within hours of the first injection of anakinra, the index patient was asymptomatic, and within days the CRP, WBC, and ferritin were all normal. IL-6 decreased with treatment as well but notably, although IL-18 was increased in patients with AOSD, it only decreased slightly relative to other cytokines. Also IL-18 bioactivity does not correlate with levels measured by enzyme-linked immunosorbent assay [15]. Withholding anakinra resulted in elevations in WBC, CRP, and ferritin again within a few days and upon restarting the IL-1 inhibitor they once again decreased. Four patients in total were successfully treated with anakinra in this initial case series. This paper suggests that the dramatic response to anakinra along with the relapse upon discontinuation, as well as previously reported efficacy of tumor necrosis factor-blocking therapies, demonstrates the involvement of IL-1 in AOSD. 

## 4. Conclusion

A presentation of a severe-appearing illness in the context of asplenia resulted in a delay of diagnosis given the concern for sepsis, particularly with an encapsulated organism. Although an infectious etiology did need to be excluded given the potential morbidity and mortality, this case represents the potential pitfalls and delays in diagnosis. It is crucial to avoid anchoring to a likely diagnosis based on context and classic associations. Although previous cases of AOSD have been documented as presenting with severe illness, this is the first reported case of an asplenic patient with AOSD masquerading as sepsis. The management of AOSD in asplenic patients will be particularly challenging, as febrile illnesses must be empirically treated with both antibiotics and anti-inflammatory agents. Such patients should be managed in careful consultation with rheumatology and infectious disease consultants.
